# Architecture of the Pancreatic Islets and Endocrine Cell Arrangement in the Embryonic Pancreas of the Grass Snake (*Natrix natrix* L.). Immunocytochemical Studies and 3D Reconstructions

**DOI:** 10.3390/ijms22147601

**Published:** 2021-07-16

**Authors:** Magdalena Kowalska, Weronika Rupik

**Affiliations:** Institute of Biology, Biotechnology and Environmental Protection, Faculty of Natural Sciences, University of Silesia in Katowice, 40-007 Katowice, Poland; magdalena.kowalska@us.edu.pl

**Keywords:** pancreatic islets, reptilian embryos, immunocytochemical studies, 3D reconstructions

## Abstract

During the early developmental stages of grass snakes, within the differentiating pancreas, cords of endocrine cells are formed. They differentiate into agglomerates of large islets flanked throughout subsequent developmental stages by small groups of endocrine cells forming islets. The islets are located within the cephalic part of the dorsal pancreas. At the end of the embryonic period, the pancreatic islet agglomerates branch off, and as a result of their remodeling, surround the splenic “bulb”. The stage of pancreatic endocrine ring formation is the first step in formation of intrasplenic islets characteristics for the adult specimens of the grass snake. The arrangement of endocrine cells within islets changes during pancreas differentiation. Initially, the core of islets formed from B and D cells is surrounded by a cluster of A cells. Subsequently, A, B, and D endocrine cells are mixed throughout the islets. Before grass snake hatching, A and B endocrine cells are intermingled within the islets, but D cells are arranged centrally. Moreover, the pancreatic polypeptide (PP) cells are not found within the embryonic pancreas of the grass snake. Variation in the proportions of different cell types, depending on the part of the pancreas, may affect the islet function—a higher proportion of glucagon cells is beneficial for insulin secretion.

## 1. Introduction

The endocrine part of the pancreas is formed by clusters of endocrine cells named pancreatic islets [1]. These islets contain four or five main types of endocrine cells: B cells—insulin-producing cells, A cells—glucagon-producing cells; D cells—somatostatin-producing cells, PP cells—pancreatic polypeptide-producing cells [2,3], and ε cells—ghrelin producing cells [4,5]. The distribution of endocrine islets within the pancreatic gland varies among different vertebrates [6]. Usually, pancreatic islets are distributed within the whole gland, as in fish [7], amphibians [8,9], birds [10], and mammals [11]. Within reptilian species, islets are distributed within the whole pancreas only in turtles [12], crocodiles [13], and some lizards [14,15]. In adult snakes, islets are distributed only in the anterior part of the pancreas, which corresponds to the dorsal pancreatic bud [16]. Thomas, 1942, presented the results based on two-dimensional (2D) histological samples that provide only partial and static information about the islet localization within the pancreas of snakes [16]. This is because these samples are usually only snapshots of distinct sections through this gland. Little is known about the distribution of endocrine tissue in the pancreas during snake embryogenesis. This study aimed to determine the spatio-temporal distribution of endocrine islets within the successive developmental stages of grass snake pancreas differentiation using 3D reconstructions. It suggested that three-dimensional reconstructions of the distribution of pancreatic islets at the successive developmental stages are an accurate method to evaluate the spatio-temporal arrangement of pancreatic islets in snakes. Moreover, this study carried out immunohistochemical localization of the endocrine cells (A, B, D, PP) within differentiating pancreatic buds. It also described the arrangement of the endocrine cells within forming islets during embryonic development. Results of this study were compared with data obtained in different vertebrate species.

## 2. Results

### 2.1. Light Microscopy

**Figure 1:**
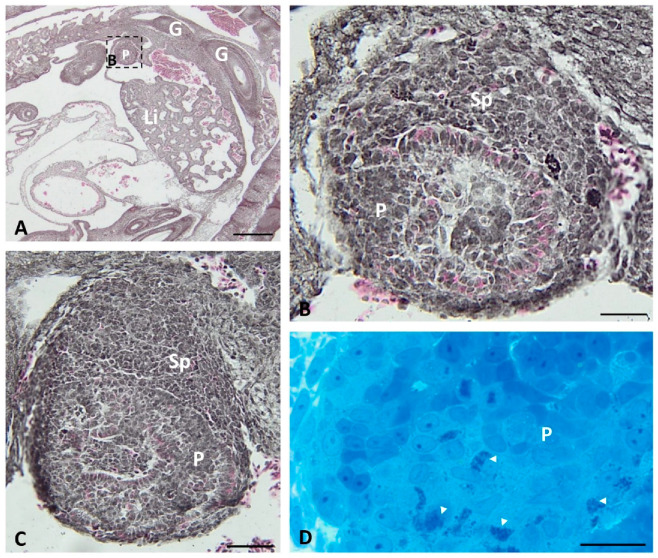
Transverse sections through the grass snake embryo body (**A**) and pancreas (**B**–**D**) at developmental stages I–III. (**A**–**C**) Sections stained with phloxine and gallocyanin. (**A**) Scale bar = 100 μm. (**B**) Magnification of the frame in (**A**). (**B**,**C**) Note agglomerations of endocrine cells near the spleen anlage. Scale bar = 20 μm. (**D**) Section stained with methylene blue. Scale bar = 20 μm Abbreviations: G: gut; Li: liver; P: pancreas; Sp: spleen; arrowhead—endocrine cell.

The presumptive endocrine pancreatic tissue is visible on the transverse histological section of the pancreas at the beginning of grass snake embryonic development (stage I–III). In the area near the spleen bud, large cords of endocrine tissue are observed (Figure 1A,B). In cords, endocrine cells stained with phloxine possess pink-staining cytoplasm near the basal pole (Figure 1C). Moreover, more caudally to this area, singular endocrine cells are visible. Within their cytoplasm, methylene blue staining agglomeration of small granules is observed (Figure 1D).

**Figure 2:**
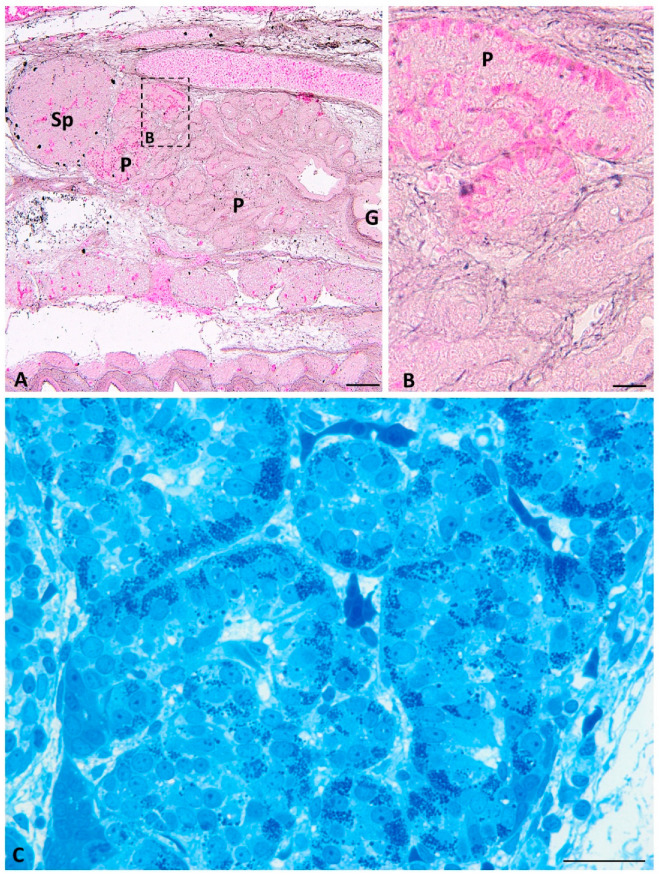
Transverse sections through the pancreas of the grass snake embryo at developmental stages IV–VIII stained with phloxine and gallocyanin (**A**,**B**) and methylene blue (**C**). (**A**) Scale bar = 100 μm. (**B**,**C**) Scale bar = 20 μm. (**B**) Magnification of the frame in (**A**). Note presumptive islets. Abbreviations: G: gut; P: pancreas; Sp: spleen.

On the longitudinal histological sections of the pancreas at developmental stages IV–VIII of grass snake embryo development, cords of endocrine tissue visible near the developing spleen are divided into presumptive islets. Within cells forming presumptive islets, the basal cytoplasm is stained with phloxine similarly to the previous developmental period (Figure 2A,B).

**Figure 3:**
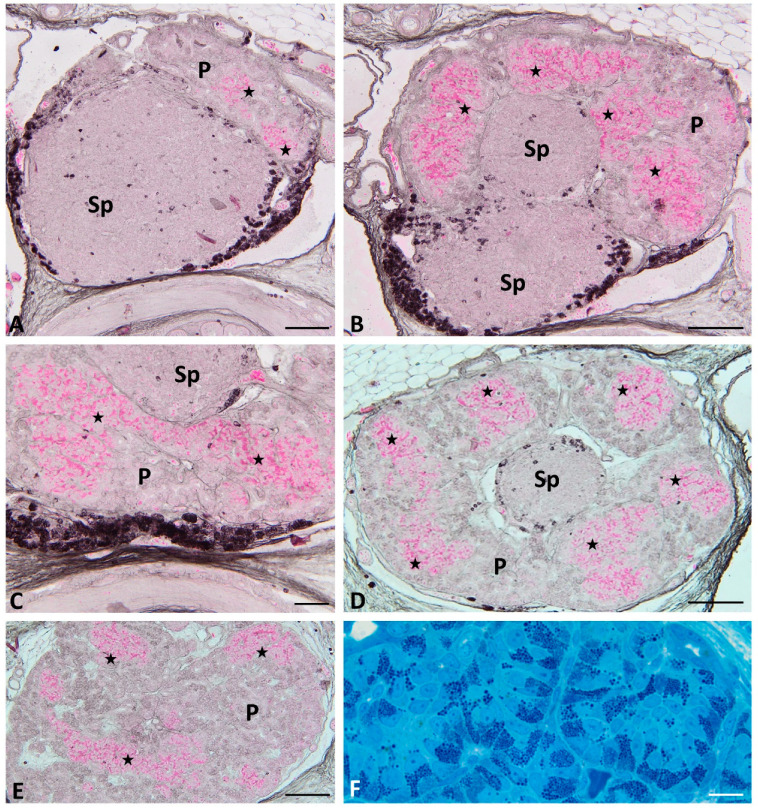
Transverse sections through the pancreas of the grass snake embryos at developmental stages IX–XI stained with phloxine and gallocyanin (**A**,**B**,**D**,**E**) and methylene blue (**F**). (**A**) Section through the most cephalic part of the pancreas. Scale bar = 100 μm. (**B**) Note splenic “bulb”. Scale bar = 100 μm. (**C**) Note connecting pancreatic islets. Scale bar = 20 μm. (**D**) Pancreatic islets forming ring around the splenic “bulb”. Scale bar = 100 μm (**E**) Small pancreatic islets within more caudal part of the dorsal pancreas. Scale bar = 100 μm. (**F**) Pancreatic islets forming large cords. Scale bar = 10 μm Abbreviations: P: pancreas; Sp: spleen; asterisk—pancreatic islets.

Moreover, on the semithin transverse sections, prominent granules are observed within the cytoplasm of cells forming islets (Figure 2C). Below the area where presumptive endocrine islets are present, pancreatic endocrine cells are not visible. On these sections through the cephalic part of the pancreas in the grass snake embryos at the last developmental period (stages IX–XI), small pancreatic islets are visible. They are located near the spleen in the pancreas process (Figure 3A). The basal cytoplasm of pancreatic endocrine cells is stained pink with phloxine, similarly as in the previous stages (Figure 3A). During this developmental period, differentiating dorsal part of the pancreas growing into the ventral part of the spleen is visible. Around this ventral part of the spleen, which is elongated, large pancreatic islets are visible. This elongated part of the spleen, surrounded by the pancreatic tissue, can be referred as the splenic “bulb” (Figure 3B).

**Figure 4:**
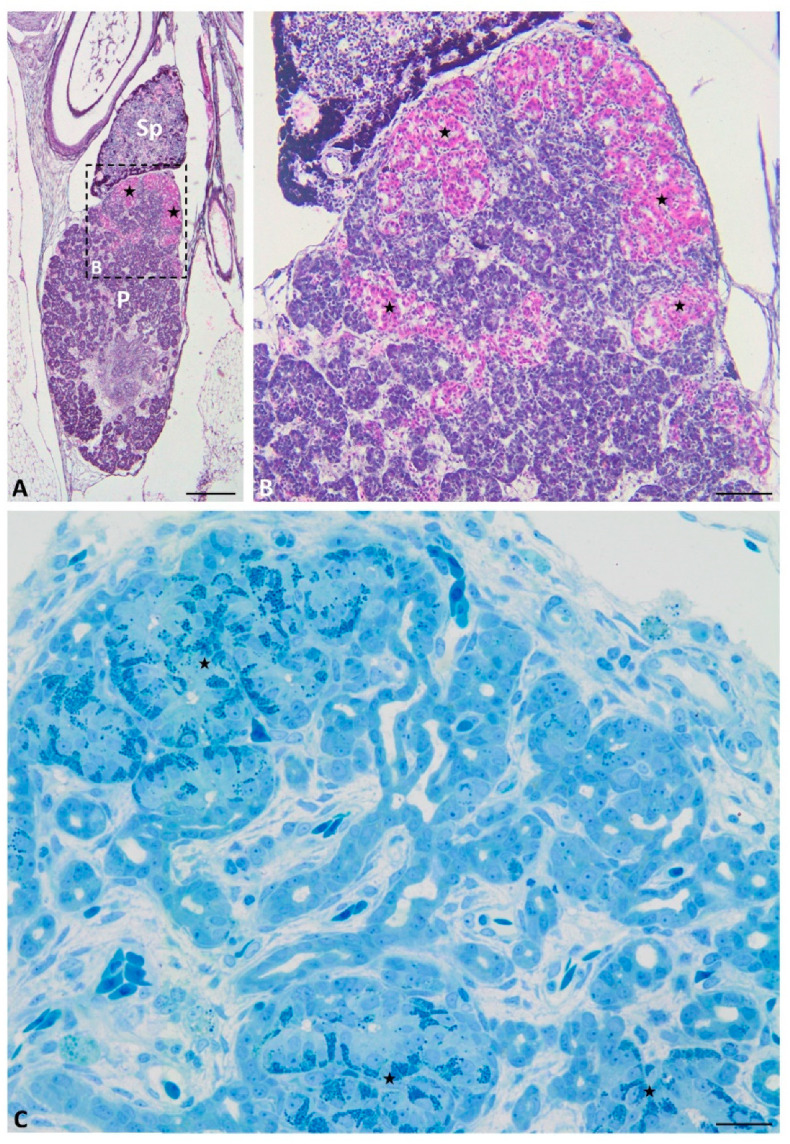
Longitudinal sections through the pancreas of the grass snake embryos at developmental stages IX–XI stained with phloxine and gallocyanin (**A**,**B**) and methylene blue (**C**). (**A**,**B**) note large pancreatic islets in the cephalic part of the dorsal pancreas. (**A**) Scale bar = 200 μm. (**B**) Scale bar = 50 μm. (**C**) Scale bar = 50 μm. Abbreviations: P: pancreas; Sp: spleen; asterisk—pancreatic islets.

Moreover, connections between large pancreatic islets are seen (Figure 3C). In semithin sections, abundant granules are observed in the basal part of the endocrine cells forming islets (Figure 3F). On the longitudinal histological sections through the pancreas, endocrine islets are present only at the cephalic part of the pancreas close to the spleen (Figure 4A,B). In these sections, islets form a “cup-like” structure on the dorsal border of the pancreas (Figure 4A,B). On the semithin longitudinal sections, abundant granules are observed in the basal part of the endocrine cells forming islets.

At the end of grass snake embryonic development (stages IX–XI), endocrine cells forming large islets are visible on the transverse histological sections throughout the dorsal part of the endocrine pancreas. They are different in shape than at the earliest stages. Large endocrine islets surrounded the spleen bulge embedded in the dorsal pancreas. They form a ring located at the periphery of the cephalic part of the dorsal pancreas (Figure 3D). Moreover, close to the caudal part of the dorsal pancreas, small groups of endocrine cells are observed (Figure 3E). They form small islets visible in the central part of this area of the pancreas. Within endocrine cells forming islets cytoplasm is stained pink with phloxine, (Figure 3E). In the longitudinal section through the endocrine pancreas, islet agglomeration formed a cup-like structure on the cephalic part of the pancreas. Below the cup-like structure, small groups of endocrine cells are visible (Figure 4A,B).

### 2.2. 3D Reconstructions

On the basis of serial paraffin sections, 3D reconstructions of endocrine islets were prepared. They showed changes in the localization and shape of pancreatic islets during the embryonic development of the grass snake.

**Figure 5:**
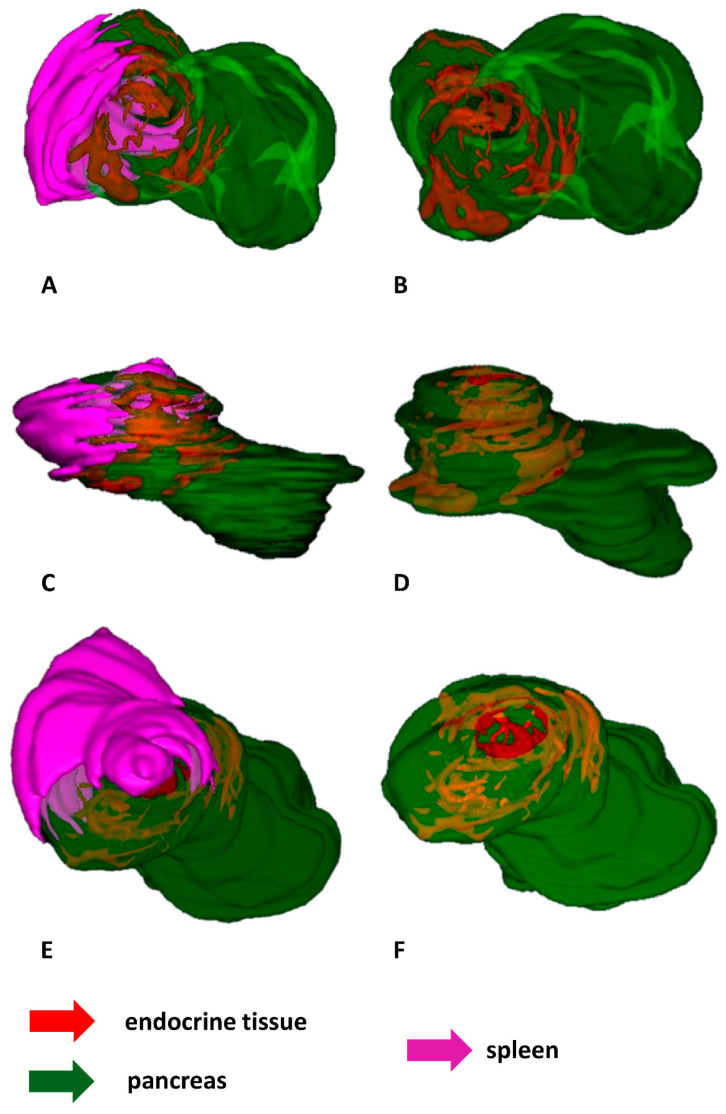
3D reconstructions of the endocrine tissue arrangement within pancreas of the grass snake embryos at developmental stages I–III. Note that endocrine tissue is localized only in the dorsal part of pancreas. (**A**) Bottom view. Pancreas with spleen visualized. (**B**) Top view. Only pancreas visualized. (**C**) Lateral view with spleen and pancreas. (**D**) Lateral view with pancreas. (**E**) Top view with spleen and pancreas. (**F**) Top view with pancreas.

During the first developmental stages of the grass snake embryos (stages I–III), endocrine tissue is visible as cords of endocrine cells within the dorsal pancreatic bud which is located near the spleen anlage. Endocrine cords are short. They are observed mainly at the periphery of the dorsal pancreatic bud, where they are oriented parallel to this bud border. Moreover, within the dorsal bud, the cords of endocrine tissue are not observed in the immediate area of the spleen anlage (Figure 5A–F).

**Figure 6:**
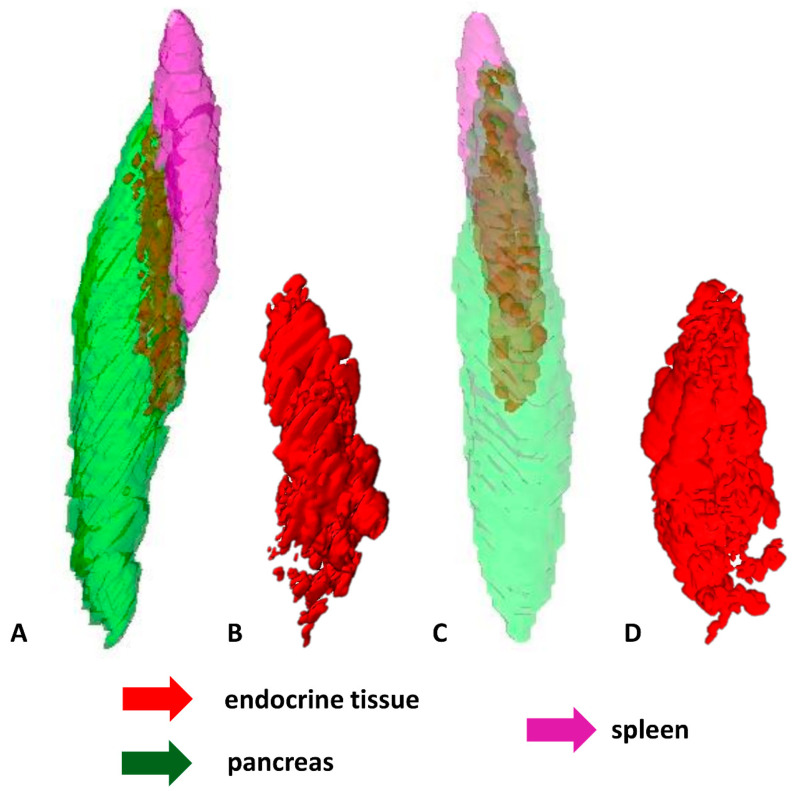
3D reconstructions of the pancreatic islet arrangement within pancreas of the grass snake embryos at developmental stages IV–VIII. Note large agglomerate of endocrine tissue. (**A**) Lateral view. Pancreas with spleen visualized. (**B**) Lateral view on pancreatic islets. (**C**) Anterio-lateral view. (**D**) Anterio-lateral view on pancreatic islets.

The shape and localization of the endocrine part of the pancreas change at the next developmental stages (stages IV–VIII). Endocrine tissue is visible as a large and elongated agglomerate of differentiating islets. It is seen at the periphery of the pancreas close to the spleen. Moreover, it is oriented parallel to the long axis of the pancreas and spleen. The agglomerate takes up approximately half the length of the pancreas. Near the prominent agglomerate of endocrine tissue, close to the central part of the pancreas, small agglomerations of endocrine tissue are visible (Figure 6A–D).

**Figure 7:**
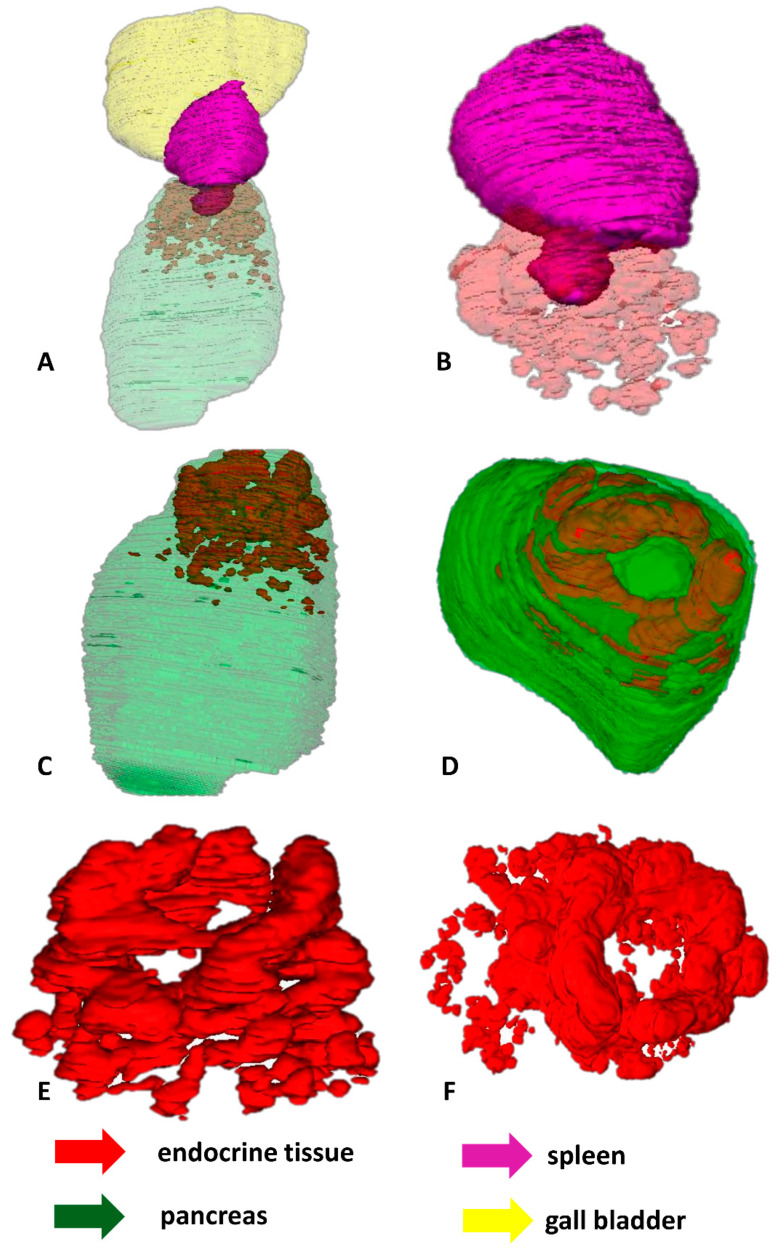
3D reconstructions of the pancreatic islet arrangement within pancreas of the grass snake embryos at developmental stages IX–XI. (**A**–**C**,**E**) lateral view, (**D**,**F**) top view. Note ring forming by pancreatic islets and splenic “bulb”.

At the end of embryonic development of the grass snake (stages IX–XI), the ventral part of the spleen forms a “bulb” embedded in the dorsal part of the pancreas. Around this “bulb”, an agglomeration of endocrine tissue in the shape of a ring is observed. The endocrine tissue forming the ring consists of smaller agglomerations of endocrine cells located at the periphery of the dorsal pancreas. These agglomerations are connected. More caudally to the major endocrine tissue agglomerations, small agglomerates of endocrine tissue are visible. The endocrine part of the pancreas is located in one-third of the pancreas (Figure 7A–F).

### 2.3. Immunofluorescence Study

**Figure 8:**
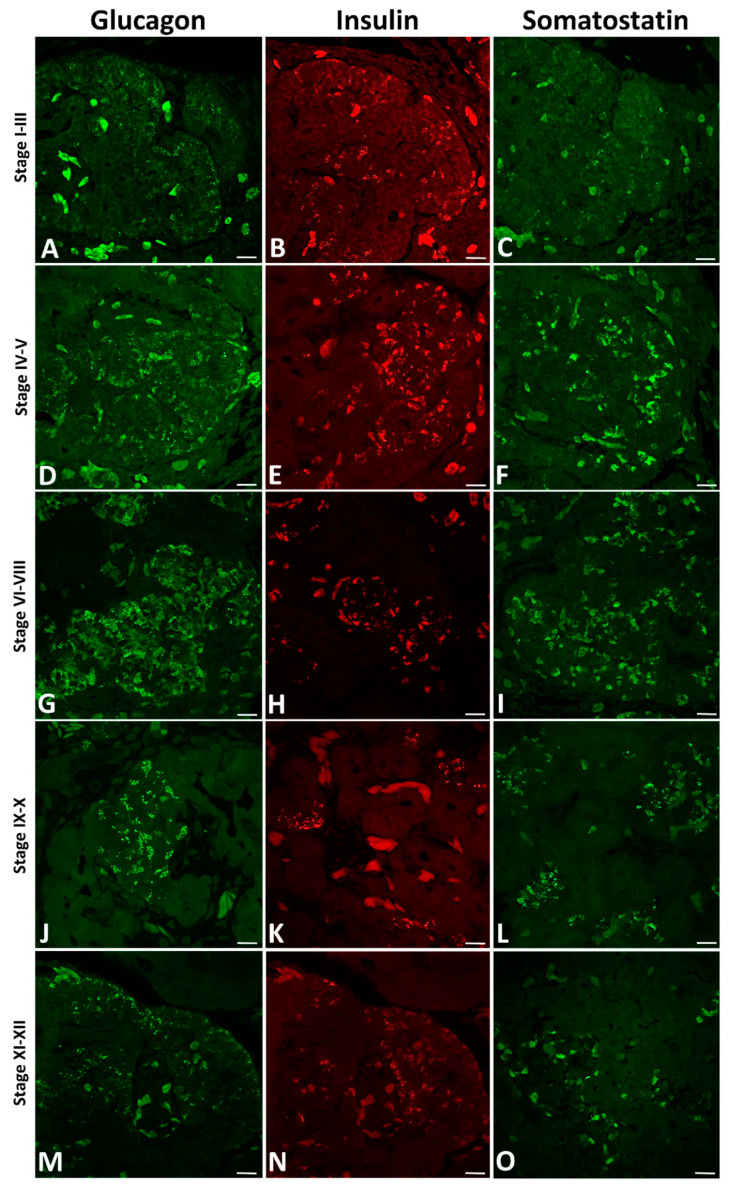
Arrangement of three main pancreatic hormones (glucagon, insulin, and somatostatin) within the pancreas of the grass snake embryos at subsequent developmental stages. (**A**,**D**,**G**,**J**,**M**) Fluorescence for glucagon. (**B**,**E**,**H,K,N**) Fluorescence for insulin. (**C**,**F**,**I**,**L**,**O**) Fluorescence for somatostatin. Scale bars—50 µm.

On the longitudinal paraffin sections of the grass snake pancreas during the early developmental period (stages I–III) immunolocalization of anti-glucagon (Figure 8A), anti-insulin (Figure 8B) and anti-somatostatin (Figure 8C) is observed. The antibody against glucagon stained many A cells located within endocrine pancreatic cords at the periphery of the dorsal bud near the spleen anlage. Single immunostained A cells near the pancreatic ducts are also visible (Figure 8A). Within the cytoplasm of A cells, glucagon-containing granules are detectable (Figure 8A). However, insulin-immunoreactive cells (B cells) are observed at both the periphery and the central part of the dorsal pancreatic bud (Figure 8B). They are visible as small groups of cells. Within the cytoplasm of B cells, tightly packed immunostained insulin-containing granules are observed (Figure 8B). Nevertheless, immunopositive somatostatin cells (D cells) are also visible (Figure 8C). They are seen randomly distributed through the dorsal pancreatic primordium (Figure 8C). Within these cells, only a few granules are observed (Figure 8C). The immunolabelling localization for all antibodies is observed to be the same localization as pink phloxine staining observed in histological sections (see Figure 1B,C).

On the longitudinal paraffin sections of the grass snake pancreas at the next developmental stages (IV–V), positive immunofluorescence signals for glucagon (Figure 8D), insulin (Figure 8E), and somatostatin (Figure 8F) stronger than earlier are visible. Immunopositive A cells are visible within small presumptive islets both at the periphery and within the central part of the dorsal pancreas. Immunolabeling for glucagon is observed on the cytoplasm of cells at the periphery of presumptive islets (Figure 8D). In addition, small groups of cells containing glucagon granules are seen near the pancreatic ducts. More abundant glucagon granules in the cytoplasm of A cells than were previously observed (Figure 8D). At the same time of grass snake embryogenesis, immunostained B cells are seen similarly as immunolabeled A cells, both at the periphery and in the central part of the dorsal pancreas (Figure 8E). However, the most accumulation of cells with insulin-positive signals is observed within presumptive islets in the cephalic part of the dorsal pancreas. Moreover, single insulin-positive fluorescence signals are seen within cells forming presumptive islets near pancreatic ducts (Figure 8E). Immunopositive fluorescence for somatostatin is observed in cells located within the entire dorsal pancreas. They are visible as groups of cells located mainly in the central part of the pancreas (Figure 8E).

During developmental stages VI to VIII of *Natrix* embryos, glucagon, insulin, and somatostatin immunolabeling is observed within the cytoplasm forming endocrine islets. All endocrine islets are observed near numerous pancreatic ducts. Glucagon-positive signals are visible within cells of irregular islets (Figure 8G). They are seen both at the periphery and the central part of islets. Insulin-positive cells are observed throughout islets which are round and elongated in shape (Figure 8H). Insulin immunolabelling is visible within the cytoplasm of cells located at the central part and the periphery of islets (Figure 8H), but at the periphery of small islets B positive cells intermingle with other endocrine cell types. Somatostatin-positive cells are seen mainly at the central part of islets, but single ones are visible at the islet periphery. They are observed among islets as large agglomerations of irregularly distributed cells (Figure 8I).

At grass snake developmental stages IX–X high immunolabelling for pancreatic hormones (glucagon, insulin, and somatostatin) is visible, as in the previous stages. However, the shape of islets formed by endocrine cells changes. Glucagon-positive cells are observed within pancreatic islets as large, oval-shaped agglomerations distributed between pancreatic ducts (Figure 8J). At the same time of embryogenesis, insulin-positive cells are seen within islets as small irregular-shaped groups near pancreatic ducts (Figure 8K). Immunoreactivity for somatostatin is observed throughout elongated groups of cells within islets. In addition, single cells immunopositive for somatostatin are observed in the central part of the dorsal pancreas (Figure 8L).

**Figure 9:**
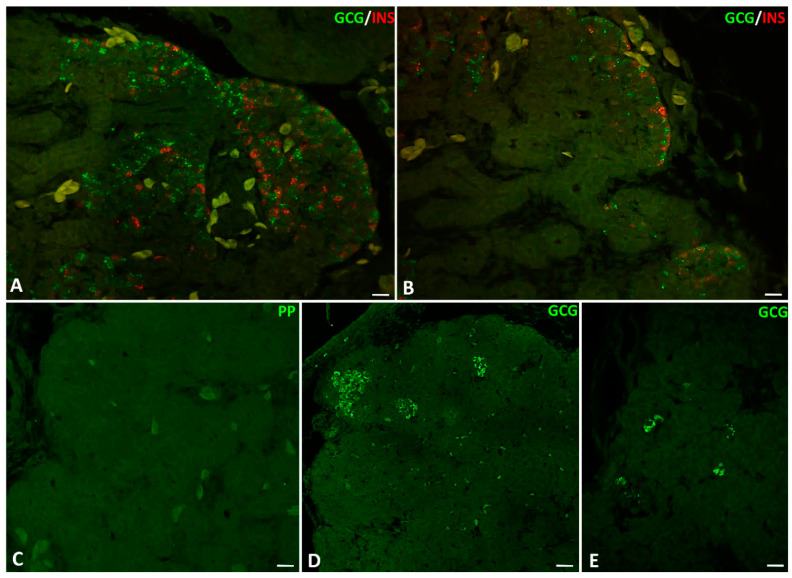
Arrangement of pancreatic endocrine hormone producing cells within pancreatic islets of the grass snake embryos at time of hatching. (**A**,**B**) Note that the A and B cells are often intermingled. (**C**) PP cells are not present within embryonic pancreas of studied species. (**D**) Large islets of A cells in more caudal part of the dorsal pancreas. (**E**) Single A cells present in the most caudal part of the dorsal pancreas. Scale bars—50 µm.

On the longitudinal sections through the pancreas of the grass snake embryos at the time of hatching, the localization of immunopositive signals for glucagon, insulin, and somatostatin is the same as in the previous stages. Immunolabelling for glucagon is observed mainly in the cytoplasm of cells within islets located at the periphery of the dorsal pancreas (Figure 8M and Figure 9A). Only single insulin-positive cells are visible in the caudal part of the dorsal pancreas (Figure 8N and Figure 9B). At this time of embryonic development, positive signals from the cytoplasm of somatostatin cells are very poor. Immunolabelling for somatostatin cells is mainly observed in the caudal part of the dorsal pancreas. These cells are visible as groups of a few cells within islets near the pancreatic ducts or solitary cells (Figure 8O). On the longitudinal sections through the dorsal pancreas, glucagon- and insulin-positive cells are often intermingled within endocrine islets. These intermingled islets containing insulin and glucagon cells are visible at the cephalic part of the dorsal pancreas (Figure 9A,B). Positive signals for insulin are mainly observed within cells located close to the blood vessels (Figure 8O and Figure 9A,B). Through all the developmental stages in the pancreas of the grass snake, positive signals for pancreatic polypeptide are not detectable (Figure 9C). Moreover, on the transverse sections through the cephalic part of the dorsal pancreas at the time of hatching, glucagon-positive cells form large islets (Figure 9D). On the other hand, on the transverse sections through the more caudal part of the dorsal pancreas, glucagon-producing cells form smaller islets (Figure 9E).

## 3. Discussion

### 3.1. Changes of Islet Localization

The organization of pancreatic endocrine tissue in many vertebrate species differs between the embryos and adult specimens [17,18]. Moreover, the structure of the pancreas changes during embryonic development. It is correlated with modifications in size, shape, and localization of the pancreatic islets at successive stages of differentiation [19,20,21,22,23,24]. The histological analysis of this study and 3D reconstructions shows that just after egg laying, the endocrine tissue in the embryonic pancreas of the grass snake is present as short cords. These cords are located within the dorsal pancreatic bud, which is apposed near the spleen anlage. Within this bud, cords appeared mainly at its periphery, parallel to the bud border. A similar appearance of cellular cords has been described in the embryonic pancreas of birds [25] and mammals [17,26,27,28]. Generally, a large mass of pancreatic endocrine tissue of vertebrates is located dorsally—precisely, within the dorsal bud-derived part of the pancreas, which is located near the spleen in adult specimens [29] as well as in embryos [18,30]. Beginning from early embryonic developmental stages, the endocrine tissue in most vertebrates is present within the dorsal part of this gland [18]. Later, during development, the endocrine tissue appears slightly within the ventral pancreatic buds [18]. In contrast, the present study shows that during the entire embryonic development of grass snake embryos, the endocrine tissue is present only in this part of the pancreas, derived from the dorsal bud. There is no evidence of the presence of the endocrine tissue in the ventral bud-derived part of the embryo, similarly as an adult pancreas in the grass snake [31,32].

The present results indicate, beginning at the next developmental period (stages IV–VIII) of the grass snake embryos, the topography and shape of the endocrine pancreas changes. From this developmental period in the pancreas of the studied species, two types of endocrine cell agglomerations are present: a large, elongated one located close to the spleen, and smaller ones localized near the central part of the body gland. The large endocrine agglomerate is oriented parallelly to the long axis of the pancreas and spleen. This organization of the endocrine tissue in the embryonic pancreas of the grass snake resembles that in the pancreas of many fish, where a large principal islet and smaller secondary islets are present [33,34,35,36]. Some authors named the large mass of endocrine tissue in the pancreas of snakes as the “principal islet” [32]. A literature review indicated that islets vary in size depending on the region of the pancreatic gland. In places derived from the ventral bud, islets are smaller, whereas in areas derived from the dorsal bud, islets are larger [11,37,38]. Moreover, larger islets are found in the region adjacent to the spleen [11], and this tendency is most marked in the pancreas of adult snakes [16,39]. It could be supposed that during embryonic development, the large cell agglomerations in the embryonic pancreas of the grass snake are formed by the connection of smaller ones. Some authors indicate that formation of a large agglomerate of endocrine tissue may be related to better paracrine signals between endocrine cells within them [38,40].

This study shows that the large agglomerate of endocrine tissue at the end of embryonic development in the grass snake (stages IX–XI) forms a ring within the cephalic part of the dorsal pancreas. This ring contains small agglomerations of endocrine cells which are connected. The formation of the pancreatic endocrine ring in the grass snake embryos is associated with the splenic “bulb” embedding in the dorsal pancreas, due to the ring surrounding the splenic “bulb.” In addition, similar to previous observations, small pancreatic islets are present within the posterior part of the dorsal pancreas. This arrangement of endocrine tissue of the grass snake embryos near the time of hatching is similar as in adult specimens of this species, where large agglomerations of islets are present in the region closest to the spleen [39,41]. It can be supposed that the stage of pancreatic endocrine ring formation in the grass snake embryos is the first step leading to the formation of intrasplenic islets in the adult specimens of this species. This is because such structures have been reported in adult specimens of *Natrix tessellata* and *Malpolon monspessulanum* [32], *Vipera berus* [42], *Vipera aspis*, and *Natrix maura* [43]. However, intrasplenic islets are not present in the juvenile specimens of above-mentioned snakes [32]. In the pancreas of juvenile specimens of the grass snake, the presence of intrasplenic islets have been not yet studied. The presence of intrasplenic islets in the pancreas of above-mentioned snakes (including representatives of family Colubridae) could be related to the fact that within their bodies, the spleen is located near the dorsal pancreas behind the gall bladder [32]. Similar close localization the spleen and the pancreas are present in the grass snake embryos.

### 3.2. Localization of Endocrine Cells within Pancreatic Islets

The present study indicates that within the embryonic pancreas of the grass snake, the first immunoreactive endocrine cells containing the main pancreatic hormones are present just after egg laying. Throughout the early embryonic life of the grass snake embryos (stages I–III), all three hormone-producing cells are detected within the cellular cords in the dorsal pancreatic bud. During this period, the somatostatin-immunopositive cells are also present, but there are far fewer of them than the other immunopositive endocrine pancreatic cells. Glucagon- and insulin-producing cells are found as small groups within cords. Their localization is similar to the endocrine cells described in chick [29,30] and mouse embryos [28,44] and human embryos [45] during the earliest stages of embryonic development.

The early presence of pancreatic hormones at detectable levels shows that their synthesis process starts long before the eggs are laid. A similar situation is also described in the lizard *Anolis carolinensis* [46]. However, quite a different situation has been described in the embryonic pancreas of *Alligator mississippiensis* [47]. Within the embryonic pancreas of this species, insulin- and glucagon-producing cells are present first, followed by somatostatin cells and then PP cells [47].

During the next developmental period (stages IV–V) of the grass snake embryos, the hormone-producing cells of the pancreas start to form presumptive islets. Within islets, immunopositive A cells are located mainly at the islet periphery, and immunopositive B cells are present at the central part of them and the islet periphery. The immunopositive D cells are detectable as small groups located only within the central part of islets. Similarly, as during the early developmental period, small groups of A cells and single B cells are located near pancreatic ducts. This study indicates that the greatest accumulation of insulin-producing cells is found in the cephalic part of the dorsal pancreas. At the same time, other pancreatic endocrine cell types are distributed more irregularly. The arrangement of endocrine cells within pancreatic islets of the grass snake embryos is similar to that in the embryonic pancreas of humans during the early developmental stages [48]. It is also somewhat similar to the pancreas of adult monkeys and humans [1]. Glucagon-producing cells in the pancreas of many vertebrates form a core-like structure surrounding the islet periphery [49].

The present results confirm that at the next developmental period (stages VI–VIII) of the grass snake embryos, pancreatic endocrine cells form islets of different shapes. Islets of glucagon-producing cells are irregular-shaped, but insulin-positive cells form round or elongated islets, whereas somatostatin-producing cells form irregular agglomerates located within islets. The hormone-producing cells are present at the central part of islets as well as at their periphery. It is worth noting that D cells are sporadically found at the periphery of islets. Moreover, all pancreatic islets are surrounded by many ducts. The arrangement of cells within pancreatic islets of the grass snake at the middle stage of embryonic development is quite different from than in the pancreas of adult *Natrix maura* and *Vipera berus*, where somatostatin cells surround the islet periphery [43].

During the later embryonic period (stages IX–X) in the pancreas of the grass snake, the shapes of endocrine islets change. It can be presumed to be the result of pancreatic endocrine tissue remodeling. The A cells start to constitute large, oval islets, but islets including B cells are small and irregular-shaped, whereas islets of D cells are small and irregular.

At the end of embryonic development of the grass snake (XI–XII), the localization of the hormone-producing cells within islets of the pancreas changes. Within the islets of the cephalic part in the dorsal pancreas, numerous insulin- and glucagon-producing cells are present, but only a few somatostatin-producing cells are found. Moreover, within the islets in the posterior part of the dorsal pancreas, more numerous glucagon- and somatostatin-producing cells, but fewer insulin-producing cells, are found. The arrangement of the endocrine cells within the islets in the dorsal pancreas differs from that in other vertebrate species. Generally, in the pancreatic regions derived from the dorsal bud of most vertebrate species, mainly in mammals, large agglomerations of glucagon cells are found, forming glucagon-rich islets [11,13]. Moreover, in the region derived from the ventral bud, significant accumulations of pancreatic polypeptide cells are present [14,50]. They form small, PP-rich islets [45,51]. Some authors reported variation in the proportions of different cell types, depending on the pancreatic part [52]. It can be hypothesized that it affects the islet function—a higher proportion of glucagon cells helps in insulin secretion. The high percentage of glucagon-producing cells in the pancreas of snakes may be related to glucose metabolism in snakes [31]. Based on the present study, glucagon-immunopositive cells form large islets within the cephalic region of the dorsal pancreas and smaller ones within their posterior part. Changes in islet size and different cellular compositions of islets are present depending on their localization within the pancreas among vertebrates. Large islets containing a high percentage of A cells in adult snakes, in general, are found in the region of the dorsal pancreas near the spleen [42]. It is also similar to other vertebrate species where the large concentration of glucagon cells in the dorsal pancreas is correlated with large A islets [11].

During hatching, A and B cells within the pancreas of the grass snake embryos are often intermingled. This endocrine cell arrangement is present at the periphery of pancreatic islets of the cephalic part of the dorsal pancreas. Moreover, a significant accumulation of insulin-positive cells is found near the blood vessels. The intermingling of the glucagon- and insulin-producing cells is similar to that in the pancreas of other snakes [41] as well as humans [40,53]. Some authors suggest that this arrangement of the A and B cells within the large islets can help B cells better react to a low concentration of blood sugar due to paracrine signaling [38,40]. A literature review indicated that in the pancreas of adult grass snakes, B cells are also arranged along the blood vessels [54]. However, it is reported that in the pancreas of many vertebrates, the endocrine cells are located along the blood vessels without specific arrangement [1].

This study indicates that during the entire embryonic development, pancreatic-polypeptide cells are not present within the pancreas of the grass snake embryos. This is similar as described in the pancreas of many adult specimens of snake species [43]. However, it is different from the pancreas of adult garter snake [41] and the pancreas of boid species [31]. Some authors suggest that the lack of pancreatic polypeptide in the snake pancreas may be related to the specialization of this organ in this group of squamates, or pancreatic polypeptide may differ in antigenicity [31].

## 4. Materials and Methods

### 4.1. Manipulation of Animals and Embryos

Studies were performed in 2017–2020 on the grass snake (*Natrix natrix*; Linnaeus, 1758), widely distributed in mainland Europe. Each year two fertilized females of *Natrix* were caught near Lubliniec and Wrocław in Poland and kept in vivaria in near-natural conditions. After egg laying, animals were released to their habitat in the place of their capture. Studies were made according to the approvals of the Regional Directorate for Environmental Protection in Katowice (WPN.6401.257.2015.DC, WPN.6401.227.2020.TL). Under Directive 2010/63/EU (Journal of Laws 2015 item 266), the study does not require the permission of the Local Ethics Committee.

Eggs of the grass snake were incubated at 30 °C and humidity of 100% in small incubators [55,56,57,58]. They were half-buried in a 1:1 mixture of sand and peat and kept in plastic storage boxes for food with transparent walls. Embryonic development of *Natrix* embryos in the laboratory conditions lasted 30–33 days [59,60]. During the developmental period, from egg laying to hatching, embryos were isolated at the same intervals of time. In each developmental stage, five embryos were used. The age of embryos was calculated based on the developmental table for this species by [61] (Table 1). The grass snake embryos were killed using a 0.25% aqueous solution of 3-aminobenzoic acid ethyl ester—MS 222, Sigma Chemical Co. (St. Louis, MO, USA) [62,63].

### 4.2. Light Microscopy

For light microscopy, whole embryos of the grass snake (stages I–III) or fragments of the body containing pancreatic tissues (stages IV–XI) were fixed in Bouin solution for two days. Then, they were dehydrated in a series of ethanol, infiltrated by xylene, paraffin I for 30 min, paraffin II for 24 h, and embedded in paraffin, according to [64]. The above-mentioned method of fixation is the best for the tissue of reptiles [65,66,67,68]. Subsequently, paraffin blocks were cut into 7 µm sections using a rotary microtome (Leica RM2125RT; Leica Biosystems, Nussloch, Germany) and collected on glass slides. Then sections were deparaffinized, stained with modified gallocyanine and phloxine methods [69], and mounted in DPX medium. Phloxine stain basal cytoplasm of pancreatic endocrine cells which contain glucagon granules. After that, sections were analyzed using an Olympus BX60 light microscope with an Olympus DP12 digital camera (Olympus, Tokyo, Japan).

The semithin sections have been obtained and stained according to the protocol which was described in [57,70].

### 4.3. 3D Reconstructions

For 3D reconstructions of the pancreatic islets, serial transverse sections stained with phloxine and gallocyanin were used. Sections were photographed using an Olympus DP12 digital camera connected to an Olympus BX60 light microscope (Olympus, Tokyo, Japan) and saved as TIFF files using CellSens Standard software. For performed reconstructions, the TrakEM2 plug-in of ImageJ [71] was used. This method was successfully applied in previous studies on the pancreas morphology during grass snake embryo development [72] and pancreatic duct differentiation [73].

### 4.4. Immunohistochemical Detection of Pancreatic Hormones

Small pieces of grass snake embryos containing the pancreas bud were fixed in a 4% paraformaldehyde solution in TBS at 4 °C overnight. After rinsing three times in TBS for 6 h each, tissues were dehydrated [74], with modifications by [75]. Dehydration followed at room temperature with 70% alcohol for 1 h, three times with 96% ethanol for 1 h, and 100% ethanol for 1 h. Then, tissues were infiltrated at room temperature three times in xylene for 50 min, paraffin I for 50 min, paraffin II for 50 min, and embedded in paraffin at 60 °C. Sections containing pancreatic tissues were cut 7 µm thick using a rotary microtome (Leica RM2125RT; Leica Biosystems, Nussloch, Germany) and collected on glass slides (SuperFrost Plus).

For the detection of pancreatic hormones (glucagon, insulin, somatostatin, and pancreatic polypeptide), antibodies produced by Santa Cruz Biotechnology Inc. were used. The following antibodies were applied: rabbit glucagon antibody (FL-180): sc-13091; mouse insulin antibody (2D11-H5): sc-8033, rabbit somatostatin antibody (H-106): sc-13099; mouse anti-pancreatic polypeptide antibody (B-2): sc-514155. The specificity and cross-reactivity of the above-mentioned antibodies were confirmed during a previous grass snake endocrine pancreatic developmental study [70]. All these antibodies were used for immunogold localization of endocrine pancreatic granules [70].

Sections were deparaffinized with xylene and rehydrated in a series of alcohol, 5 min each. Then, they were incubated in 0.3% H_2_O_2_ in TBS for 15 min, followed by rinsing three times in TBS, 5 min each. After this, sections were washed in TBS with 0.1% Triton X-100 for 15 min and incubated in 2% BSA in TBS for 1 h at room temperature to block endogenous peroxidase. Then, sections were incubated with the primary antibodies in 2% BSA at 4 °C overnight at the following dilutions: anti-glucagon (1:400), anti-insulin (1:100), anti-somatostatin (1:400), and anti-pancreatic polypeptide (1:50). In the control sections, primary antibodies were omitted. After rinsing three times in TBS for 5 min each, sections were incubated in the secondary antibodies: goat anti-rabbit IgG (H + L), cross-adsorbed secondary antibody, Alexa Fluor 488 (1:800); goat anti-mouse IgG (H + L) cross-adsorbed secondary antibody, Alexa Fluor 594 (1:200). Then, sections were rinsed three times in TBS, 5 min each, and counterstained with DAPI for 15 min (1 mg/mL of an aqueous solution diluted 1: 1000 in absolute ethanol). After that, sections were washed three times in TBS for 5 min and mounted in glycerine ≥99.5% for fluorescence microscopy (Sigma-Aldrich). For double immunofluorescence studies, sections were incubated simultaneously in a mixture of two primary antibodies (mouse anti-glucagon—1:400 and rabbit anti-insulin—1:100) and then in a combination of secondary antibodies (anti-mouse Alexa Fluor 488—1:800 and anti-rabbit Alexa Fluor 546—1:200). Prepared slides were kept at 4 °C in the dark before being analyzed. Samples were analyzed using an Olympus IX81 inverted microscope equipped with the Olympus FLUOVIEW FV1000 confocal laser system.

## 5. Conclusions

Differentiation of the pancreatic endocrine cellular cords in the grass snake embryos leads to the formation of a ring surrounding the splenic “bulb”. Consequently, intrasplenic islets, characteristic for adult specimens of a few snakes, for example *Natrix* species, may be formed. The presence of intrasplenic islets could be related to the location of the spleen near the dorsal pancreas. In addition to this differentiation of the endocrine part of the pancreas in the studied species at the time of hatching, in the cephalic part of the dorsal pancreas, large A islets are found. The high percentage of A cells could be connected to the metabolism of glucose in snakes. The great content of glucagon cells can help the secretion of insulin. Moreover, in the pancreas of the studied species, A cells are intermingled with B cells at the periphery of islets. This arrangement of endocrine cells can lead to the better reaction of B cells, paracrine signaling, and a low concentration of blood sugar. It is worth noting that, in the present study, pancreatic polypeptide cells were not found in the embryonic pancreas of the grass snake. The lack of PP cells may be related to the specialization of the pancreas in this group of squamates or different antigenicity.

## Figures and Tables

**Table 1:** The relationship between incubation time and embryo body size [61].

Incubation Time (Days)	Total Length (mm)	Head Length (mm)	Head Width (mm)	Head Height (mm)	Tail Length (mm)	Embryo Age Defined by Stages
0	26.50	2.30	1.40	3.90	No	I
1	27.00	2.50	1.60	4.40	No	
2	30.00	3.20	1.80	4.80	No	II
3	31.50	3.70	1.80	4.90	2.00	
4	32.10	3.90	2.00	4.50	3.00	III
5	34.90	3.90	2.10	4.30	4.30	IV
6	43.80	4.10	2.20	4.20	5.20	
7	48.70	4.50	2.40	4.20	5.70	V
8	58.50	4.80	2.40	4.00	7.80	
9	79.30	4.80	2.50	4.00	9.20	
10	82.70	5.20	2.70	3.80	11.30	VI
12	87.60	6.40	2.70	3.70	11.90	
14	98.20	7.10	2.90	3.70	15.80	VII
17	133.70	8.00	3.30	3.50	17.10	VIII
20	144.20	8.30	3.80	3.30	25.90	IX
22	153.10	8.70	3.90	3.20	29.30	X
25	176.30	9.40	4.00	3.00	30.70	XI
27	184.70	11.30	4.20	3.00	34.20	
31	200.00	11.80	4.70	3.00	38.50	XII
32	211.30	12.00	4.70	3.00	40.00	
33	211.80	12.00	4.70	3.00	40.10	

## Data Availability

Not applicable.

## References

[1] Cabrera O., Berman-Weinberg D.M., Kenyon N.S., Ricordi C., Berggren P.-O., Caicedo A. (2006). The unique cytoarchitecture of human pancreatic islets has implications for islet cell function. Proc. Natl. Acad. Sci. USA.

[2] Slack J.M. (1995). Developmental biology of the pancreas. Development.

[3] Gittes G.K. (2009). Developmental biology of the pancreas: A comprehensive review. Dev. Biol..

[4] Wierup N., Svensson H., Mulder H., Sundler F. (2002). The ghrelin cell: A novel developmentally regulated islet cell in the human pancreas. Regul. Pept..

[5] Napolitano T., Silvano S., Vieira A., Balaji S., Garrido-Utrilla A., Friano M.E., Atlija J., Collombat P. (2018). Role of ghrelin in pancreatic development and function. Diabetes Obes. Metab..

[6] Ionescu-Tirgoviste C., Gagniuc P.A., Gubceac E., Mardare L., Popescu I., Dima S., Militaru M. (2015). A 3D map of the islet routes throughout the healthy human pancreas. Sci. Rep..

[7] Youson J.H., Al-Mahrouki A.A., Amemiya Y., Graham L.C., Montpetit C.J., Irwin D.M. (2006). The fish endocrine pancreas: Review, new data, and future research directions in ontogeny and phylogeny. Gen. Comp. Endocrinol..

[8] El-Salhy M., Wilander E., Abu-Sinna G. (1982). The endocrine pancreas of anuran amphibians: A histological and immunocytochemical study. Biomed. Res..

[9] Francini F., Madsen O., Dumm C.L.G., Gagliardino J.J. (1996). Topographic Differences in Cell Populations and Insulin Secretion in the Endocrine Pancreas of the Toad *Bufo arenarum*. Gen. Comp. Endocrinol..

[10] Dupont J., Rideau N., Simon J., Scanes C.G. (2015). Chapter 27—Endocrine Pancreas. Sturkie’s Avian Physiology.

[11] Elayat A.A., El-Naggar M.M., Tahir M. (1995). An immunocytochemical and morphometric study of the rat pancreatic islets. J. Anat..

[12] Epple A., Brinn J.E. (1987). The Comparative Physiology of the Pancreatic Islets.

[13] Rhoten W.B. (1987). Quantitative immunocytochemical analysis of the endocrine pancreas of the Nile crocodile. Am. J. Anat..

[14] El-Salhy M., Grimelius L. (1981). Histological and immunohistochemical studies of the endocrine pancreas of lizards. Histochemistry.

[15] El-Salhy M., Abu-Sinna G., Wilander E. (1983). The endocrine pancreas of a squamate reptile, the desert lizard (*Chalcides ocellatus*). Histochemistry.

[16] Thomas T.B. (1942). The pancreas of snakes. Anat. Rec. Adv. Integr. Anat. Evol. Biol..

[17] Björkman N., Hellerström C., Hellman B., Petersson B. (1966). The cell types in the endocrine pancreas of the human fetus. Z. Zellforsch. Mikrosk. Anat..

[18] Maňáková E., Titlbach M. (2007). Development of the Chick Pancreas with Regard to Estimation of the Relative Occurrence and Growth of Endocrine Tissue. Anat. Histol. Embryol..

[19] Kaung H.-L.C. (1981). Immunocytochemical localization of pancreatic endocrine cells in frog embryos and young larvae. Gen. Comp. Endocrinol..

[20] Mikami S.-I., Sudo S., Taniguchi K., Yamada S. (1986). Immunocytochemical studies on the development of the pancreatic islet cells in the domestic fowl. Jpn. J. Vet. Sci..

[21] Reddy S., Bibby N.J., Elliott R.B. (1992). An immunocytochemical study of endocrine cell development in the early fetal guinea pig pancreas. Gen. Comp. Endocrinol..

[22] Bricout-Neveu E., Pechberty S., Reynaud K., Maenhoudt C., José Lecomte M., Ravassard P., Czernichow P. (2017). Development of the Endocrine Pancreas in the Beagle Dog: From Fetal to Adult Life. Anat. Rec. Adv. Integr. Anat. Evol. Biol..

[23] Gupta D., Uppal V., Bansal N., Gupta A. (2020). Differentiation of pancreatic endocrine islets in buffalo fetus. Indian J. Anim. Sci..

[24] Nagaya M., Hayashi A., Nakano K., Honda M., Hasegawa K., Okamoto K., Itazaki S., Matsunari H., Watanabe M., Umeyama K. (2019). Distributions of endocrine cell clusters during porcine pancreatic development. PLoS ONE.

[25] Da Silva A.B.S., Fonseca C.M.B., Cavalcante M.M.A.D.S., De Oliveira I.M., Ferraz M.S., Viana F.J.C., Fontenele R.D., Conde Júnior A.M. (2018). Histomorphometry of pancreas development in hybrid chicken (*Galus galus*) embryo and fetus. Microsc. Res. Tech..

[26] Hara A., Kadoya Y., Kojima I., Yamashina S. (2007). Rat pancreatic islet is formed by unification of multiple endocrine cell clusters. Dev. Dyn..

[27] Jeon J., Correa-Medina M., Ricordi C., Edlund H., Diez J.A. (2009). Endocrine Cell Clustering During Human Pancreas Development. J. Histochem. Cytochem..

[28] Miller K., Kim A., Kilimnik G., Jo J., Moka U., Periwal V., Hara M. (2009). Islet Formation during the Neonatal Development in Mice. PLoS ONE.

[29] Şimşek N., Bayraktaroğlu A.G., Altunay H. (2009). Localization of insulin immunpositive cells and histochemical structure of the pancreas in falcons (*Falco naumanni*). Ankara Üniv. Vet. Fak. Derg..

[30] Rawdon B.B. (1998). Morphogenesis and differentiation of the avian endocrine pancreas, with particular reference to experimental studies on the chick embryo. Microsc. Res. Tech..

[31] Buchan A.M.J. (1984). An immunocytochemical study of endocrine pancreas of snakes. Cell Tissue Res..

[32] Moscona A.A. (1990). Anatomy of the pancreas and langerhans islets in snakes and lizards. Anat. Rec. Adv. Integr. Anat. Evol. Biol..

[33] Youson J.H., Al-Mahrouki A.A. (1999). Ontogenetic and Phylogenetic Development of the Endocrine Pancreas (Islet Organ) in Fishes. Gen. Comp. Endocrinol..

[34] Lee J.H., Ku S.K., Park K.D., Lee H.S. (2001). Comparative study of endocrine cells in the principal pancreatic islets of two teleosts, *Silurus asotus* (Siluridae) and *Siniperca scherzeri* (Centropomidae). J. Vet. Sci..

[35] Li Z., Wen C., Peng J., Korzh V., Gong Z. (2009). Generation of living color transgenic zebrafish to trace somatostatin-expressing cells and endocrine pancreas organization. Differentiation.

[36] Fortin J.S., Santamaria-Bouvier A., Lair S., Dallaire A.D., Benoit-Biancamano M.-O. (2015). Anatomic and molecular characterization of the endocrine pancreas of a teleostean fish: Atlantic wolffish (*Anarhichas lupus*). Zool. Stud..

[37] Saito K., Iwama N., Takahashi T. (1978). Morphometrical analysis on topographical difference in size distribution, number and volume of islets in the human pancreas. Tohoku J. Exp. Med..

[38] Ravi P.K., Purkait S., Agrawal U., Patra S., Patnaik M., Singh S.R., Mishra P.R. (2019). Regional variation of human pancreatic islets dimension and its impact on beta cells in Indian population. Islets.

[39] Miller M.R. (1962). Observations on the comparative histology of the reptilian pancreatic islet. Gen. Comp. Endocrinol..

[40] Kilimnik G., Jo J., Periwal V., Zielinski M., Hara M. (2012). Quantification of islet size and architecture. Islets.

[41] Rhoten W.B. (1984). Immunocytochemical localization of four hormones in the pancreas of the garter snake, *Thamnophis sirtalis*. Anat. Rec. Adv. Integr. Anat. Evol. Biol..

[42] Hellerström C., Asplund K. (1966). The two types of A-cells in the pancreatic islets of snakes. Z. Zellforsch. Mikrosk. Anat..

[43] Masini M.A. (1988). Immunocytochemical localization of peptides in the endocrine pancreas of the snakes *Vipera aspis* and *Natrix maura*. Acta Histochem..

[44] Teitelman G., Joh T.H., Reis D.J. (1981). Transformation of catecholaminergic precursors into glucagon (A) cells in mouse embryonic pancreas. Proc. Natl. Acad. Sci. USA.

[45] Proshchina A.E., Krivova Y., Barabanov V.M., Saveliev S.V. (2019). Pancreatic endocrine cell arrangement during human ontogeny. Acta Histochem..

[46] Rhoten W.B., Hall C.E. (1982). An immunocytochemical study of the cytogenesis of pancreatic endocrine cells in the lizard, *Anolis carolinensis*. Am. J. Anat..

[47] Jackintell L.A., Lance V.A. (1994). Ontogeny and Regional Distribution of Hormone-Producing Cells in the Embryonic Pancreas of *Alligator mississippiensis*. Gen. Comp. Endocrinol..

[48] Robb P. (1961). The development of the islets of Langerhans in the human foetus. Q. J. Exp. Physiol. Cogn. Med Sci..

[49] Steiner D.J., Kim A., Miller K., Hara M. (2010). Pancreatic islet plasticity: Interspecies comparison of islet architecture and composition. Islets.

[50] Dolenšek J., Rupnik M.S., Stožer A. (2015). Structural similarities and differences between the human and the mouse pancreas. Islets.

[51] Baetens D., Malaisse-Lagae F., Perrelet A., Orci L. (1979). Endocrine pancreas: Three-dimensional reconstruction shows two types of islets of langerhans. Science.

[52] Trimble E.R., Halban P.A., Wollheim C.B., Renold A.E. (1982). Functional differences between rat islets of ventral and dorsal pan-creatic origin. J. Clin. Investig..

[53] Moede T., Leibiger I.B., Berggren P.-O. (2020). Alpha cell regulation of beta cell function. Diabetologia.

[54] Trandaburu T., Calugareanu L. (1969). Light and electron microscopic investigation of the endocrine pancreas of the grass-snake [*Natrix n. natrix* (L.)]. Z. Zellforsch. Mikrosk. Anat..

[55] Rupik W. (2012). Hollowing or cavitation during follicular lumen formation in the differentiating thyroid of grass snake *Natrix natrix* L. (Lepidosauria, Serpentes) embryos? An ultrastructural study. Zoology.

[56] Rupik W. (2013). Ultrastructural studies of cilia formation during thyroid gland differentiation in grass snake embryos. Micron.

[57] Kowalska M., Rupik W. (2020). Development of pancreatic acini in embryos of the grass snake *Natrix natrix* (Lepidosauria, Serpentes). J. Morphol..

[58] Kaczmarek P., Rupik W. (2021). Structural and ultrastructural studies on the developing vomeronasal sensory epithelium in the grass snake *Natrix natrix* (Squamata: Colubroidea). J. Morphol..

[59] Kaczmarek P., Hermyt M., Rupik W. (2017). Embryology of the VNO and associated structures in the grass snake *Natrix natrix* (Squamata: Natricinae): A 3D perspective. Front. Zool..

[60] Kowalska M., Rupik W. (2018). Ultrastructure of endocrine pancreatic granules during pancreatic differentiation in the grass snake, *Natrix natrix* L. (Lepidosauria, Serpentes). J. Morphol..

[61] Rupik W. (2002). Early development of the adrenal glands in the grass snake *Natrix natrix* L. (Lepidosauria, Serpentes). Adv. Anat. Embryol. Cell Biol..

[62] Baier J. (2006). Reptiles. Guidelines for the Euthanasia of Non-Domestic Animals.

[63] Conroy C.J., Papenfuss T., Parker J., Hahn N.E. (2009). Use of Tricaine Methanesulfonate (MS222) for Euthanasia of Reptiles. J. Am. Assoc. Lab. Anim. Sci..

[64] Bagiński S. (1969). Technika Mikroskopowa.

[65] Rupik W., Swadźba E., Dubińska-Magiera M., Jędrzejowska I., Daczewska M. (2012). Reptilian myotomal myogenesis—Lessons from the sand lizard *Lacerta agilis* L. (Reptilia, Lacertidae). Zoology.

[66] Rupik W., Kowalska M., Swadźba E., Maślak R. (2016). Ultrastructural features of the differentiating thyroid primordium in the sand lizard (*Lacerta agilis* L.) from the differentiation of the cellular cords to the formation of the follicular lumen. Zoology.

[67] Kaczmarek P., Janiszewska K., Metscher B., Rupik W. (2020). Development of the squamate naso-palatal complex: Detailed 3D analysis of the vomeronasal organ and nasal cavity in the brown anole *Anolis sagrei* (Squamata: Iguania). Front. Zool..

[68] Kaczmarek P., Metscher B., Rupik W. (2021). Embryology of the naso-palatal complex in Gekkota based on detailed 3D analysis in *Lepidodactylus lugubris* and *Eublepharis macularius*. J. Anat..

[69] Zadorozhnyĭ V.V. (1982). Modified histological methods using a nuclear dye gallocyanine. Arkh. Patol..

[70] Kowalska M., Rupik W. (2019). Development of endocrine pancreatic islets in embryos of the grass snake *Natrix natrix* (Lepidosauria, Serpentes). J. Morphol..

[71] Rasband W.S. (1997–2016). ImageJ. U.S..

[72] Kowalska M., Hermyt M., Rupik W. (2017). Three-dimensional reconstruction of the embryonic pancreas in the grass snake *Natrix natrix* L. (Lepidosauria, Serpentes) based on histological studies. Zoology.

[73] Kowalska M., Rupik W. (2018). Development of the duct system during exocrine pancreas differentiation in the grass snake *Natrix natrix* (Lepidosauria, Serpentes). J. Morphol..

[74] Walter I., Fleischmann M., Klein D., Müller M., Salmons B., Günzburg W.H., Renner M., Gelbmann W. (2000). Rapid and sensitive detection of enhanced green fluorescent protein expression in paraffin sections by confocal laser scanning microscopy. Histochem. J..

[75] Rupik W., Stawierej A., Stolarczyk I., Widłak W. (2006). Promoter of the heat shock testis-specific Hsp70.2/Hst70 gene is active in nervous system during embryonic development of mice. Anat. Embryol..

